# Fever Phobia as a Reason for Pediatric Emergency Department Visits: Does the Primary Care Physician Make a Difference?

**DOI:** 10.5041/RMMJ.10282

**Published:** 2017-01-30

**Authors:** Erella Elkon-Tamir, Ayelet Rimon, Dennis Scolnik, Miguel Glatstein

**Affiliations:** 1 Pediatric Emergency Medicine, Dana-Dwek Children’s Hospital, Tel Aviv Medical Center, Sackler Faculty of Medicine, Tel Aviv University, Israel; 2 Pediatric Emergency Medicine and Clinical Pharmacology and Toxicology, The Hospital for Sick Children, University of Toronto, Ontario, Canada; 3 Clinical Pharmacology and Toxicology, Tel Aviv Sourasky Medical Center, Sackler Faculty of Medicine, Tel Aviv University, Israel

**Keywords:** Antipyretic, anxiety, fever, fever phobia, parent

## Abstract

**Background:**

Fever is a source of considerable parental anxiety. Numerous studies have also confirmed similar anxiety among health care workers. This study analyzed caregiver knowledge of fever, and beliefs concerning children with a febrile illness, with an emphasis on the referring physician.

**Methods:**

This was a cross-sectional study of 100 caregivers of children 3 months to 12 years old, treated at an urban tertiary care pediatric emergency department for fever. Caregiver knowledge was assessed with a questionnaire.

**Results:**

Most caregivers correctly defined the threshold for fever as >38.0–38.3°C. Caregivers commonly believed that fever can cause brain damage and epilepsy; the frequency of this belief was not affected by whether they were referred to the emergency department by their pediatrician/family physician or by another physician or arrived without a referral. For a comfortable-appearing child with a temperature not above 38.0°C, both groups reported that they would give antipyretics in similar proportions (mean 31%). The majority of parents in both groups believed that teething could cause fever (mean 74%).

**Conclusion:**

Caregivers in this study had limited knowledge of fever and its management in children, even if referred by their primary care physician. We suggest that there is a need for aggressive educational interventions to reduce parents’ fever phobia, in clinics as well as in pediatric emergency departments, and that this need may extend to the education of medical personnel as well.

## BACKGROUND

Fever is the chief complaint in up to one-third of all pediatric office visits.^1^ The pathophysiology of this condition is relatively well defined. Body temperature is regulated by thermo-sensitive neurons, located in the pre-optic or anterior hypothalamus, that respond to change in blood temperature as well as to direct neural connections with cold and warm receptors located in skin and muscle.^1^,^2^ The body does not allow lethal temperature to occur as long as there is no dehydration and an open environment is provided to allow for heat loss.^3^,^4^ The rare exception to this is when there is an underlying neurological condition affecting the temperature control center (e.g. hypothalamic lesion). Furthermore, fever is purposeful but the effects of its reduction on disease duration and severity are questionable.^5^,^6^

Fever phobia is a persistent problem, and care-givers continue to be anxious when dealing with febrile children, their main concerns being possible central nervous system damage (24%), seizures (19%), and death (5%).^3^ Dr Barton Schmitt coined the term “fever phobia” in 1980.^7^ He found that almost all parents believe that fever can cause physical harm to their children, despite the reality that fever is a physiologic process and not a primary illness in itself. The majority of parents surveyed reported that their views on fever were mostly influenced by what they learned in the physician’s office. Many caregivers also believe that, without treatment, temperature can rise to harmful levels.^8^ Without a clear understanding of their child’s fever, many parents often head straight to the medicine cabinet, not realizing that an elevation in temperature is a complex immune response that may actually help the child fight off the infection.^8^

Fever is among the most common topics discussed by pediatricians and parents, and it is clear that health care workers need to follow rational guidelines in investigating, identifying, and treating serious causes of fever, but it is not clear that health care workers make the necessary effort to decrease the degree of fever phobia.^9^ Current practice of community physicians in Israel does not include handing out written information about fever management to the parents.

Assessment of parental ability to correctly define clinically meaningful temperature ranges in children, and assessment of their beliefs regarding fever, may facilitate a better understanding of the knowledge gap between clinicians and parents regarding fever. The primary aim of this study was to compare knowledge regarding fever in two groups of parents bringing their children to a pediatric emergency department (ED): those referred by a pediatrician or a family physician versus those self-referred, and to asses where educational initiatives can best be directed.

## METHODS

A convenience sample of caregivers of children with a febrile illness, brought to the pediatric ED of an urban tertiary care medical center in Tel Aviv, Israel, during May and August 2014, was recruited. Care-givers of children who were admitted were excluded from the study. During their stay in the ED, the caregivers completed a one-page questionnaire. Questions included definition of fever, the presumed consequences of fever, the use of antipyretics, and investigations deemed necessary.

Following the data collection, the caregivers were evaluated as two groups: Group 1 consisted of care-givers who brought the child to the ED with a referral from their primary care physician (pediatrician or family physician), and Group 2 consisted of families without referrals and children that were referred by a physician other than the primary care physician or by a medical call center.

The data were analyzed using SAS for Windows Version 9.2. Univariate analysis was used to characterize the examined variables by the source of referral to ED: pediatrician/family physician, or other. Continuous variables were reported by means and standard deviations, or by the median and interquartile range, depending on their distribution (normal or abnormal, respectively). Categorical variables were reported by their relative abundances. The Pearson chi-square test or Fisher’s exact test was used to compare the groups with respect to categorical variables. Two-sample *t* test was used to compare the two groups with respect to variables that follow a normal distribution. Two-sample Wilcoxon test was used to compare the two groups with respect to variables that do not follow a normal distribution.

The study was approved by the institutional research ethics committee.

## RESULTS

A total of 100 caregivers completed the questionnaire; 60% were referred by their primary care physician (Group 1), and 40% were referred by a physician other than their primary care physician, by a medical call center, or had no referral (Group 2). Both groups’ demographics and previous clinical experience of participants were comparable (Table 1). In addition to most of the patients in Group 1 (92%), a large number (40%) of the patients in Group 2 had been examined by their pediatrician since the current illness started.

**Table 1:** Demographics of Participants.

Variables	Total (*n*=100)	Group 1 (*n*=60)	Group 2 (*n*=40)	P*
Age of child, months				
Median	18.5	18	25	0.27
Range	3-143	3-126	6-143	

Relationship of participant to child, *n* (%)				
Father	24 (24)	14 (23)	10 (25)	1.0
Mother	76 (76)	46 (77)	30 (75)	

Age of parent, years				
Mean	35.7	35.8	35.7	0.96
Range	19-54	27-49	19-54	

Education of parent, *n* (%)				
High school graduate	100 (100)	60 (100)	40 (100)	0.1
University graduate	75 (75)	49 (82)	26 (65)	

Number of children in family, median (IQR)	2 (1, 3)	2 (1, 3)	2 (2, 3)	0.41

Child is firstborn, *n* (%)	55 (55)	35 (58)	20 (50)	0.54

Influencing factors, *n* (%)				
Epilepsy in family	3 (3)	1 (2)	2 (5)	0.56
Febrile seizures in family	13 (13)	4 (7)	9 (23)	0.045
Child examined by regular pediatrician during the present illness, *n* (%)	71 (71)	55 (92)	16 (40)	<0.001

* Wilcoxon two-sample test, *t* test, or chi-square comparison of two groups.

Fever was the chief complaint in 86% of cases (Table 2). The body temperature was measured using a thermometer at home in 97% of the children. The most prevalent response to the definition of the threshold of fever was the correct response (38–38.3°C); however, 16% of caregivers defined the fever threshold at a temperature less than 38°C (Table 3). There was no difference in the definition of fever between the two groups. Likewise, common beliefs regarding fever did not differ significantly between the two groups (Table 4): 45% of caregivers believed that fever could cause brain damage, and 74% believed that teething caused fever. Thirty-one percent of caregivers said they would give antipyretics to a comfortable-appearing child with a temperature of 38°C, and 10% would give antipyretics to a comfortable-appearing child with a temperature between 37.4 and 37.8°C. Twenty-five percent of caregivers felt that a febrile child should always be examined by a physician.

**Table 2:** Reason for ED Visit.

Chief Complaint, *n* (%)*	Total (*n*=100)	Group 1 (*n*=60)	Group 2 (*n*=40)	P**
Fever	86 (86)	52 (87)	34 (85)	1.0

Febrile seizure	3 (3)	0 (0)	3 (7)	0.06

Vomiting / Diarrhea	9 (9)	4 (7)	5 (13)	0.48

Rash	3 (3)	3 (5)	0 (0)	0.27

Cough / Shortness of breath	12 (12)	9 (15)	3 (7)	0.35

Other	29 (29)	17 (28)	12 (30)	1.0

Method of temperature measurement at home, *n* (%)				0.56
Thermometer	97 (97)	59 (98)	38 (95)	
Tactile	3 (3)	1 (2)	2 (5)	

* More than one complaint per survey possible.

** Fisher’s exact test or chi-square comparison of two groups.

**Table 3:** Definition of Fever Reported by Caregivers.

	Total (*n*=100)	Group 1 (*n*=60)	Group 2 (*n*=40)	P*
Definition of fever threshold °C, *n* (%)				>0.06
37.0–37.5	2 (2)	2 (3)	0 (0)	
37.5–37.9	14 (14)	8 (13)	6 (15)	
38.0–38.3	45 (45)	24 (40)	21 (53)	
38.3–38.5	15 (15)	7 (12)	8 (20)	
38.5–39.0	23 (23)	19 (32)	4 (10)	
≥39.0	1 (1)	0 (0)	1 (3)	

* Fisher’s exact test.

**Table 4:** Fever-Related Beliefs and Behaviors.

Variables, *n* (%)	Total (*n*=100)	Group 1 (*n*=60)	Group 2 (*n*=40)	P*	CI
Fever causes brain damage	45 (45)	24 (40)	21 (53)	0.2	−8.22 to 33.22
Fever causes epilepsy	16 (16)	10 (17)	6 (15)	0.79	−15.08 to 17.10
Would give antipyretics to a comfortable child with temperature of 38.0°C	31 (31)	17 (28)	14 (35)	0.46	−12.44 to 26.89
Would give antipyretics to a comfortable child with temperature 37.4°C to 37.8°C	10 (10)	7 (12)	3 (8)	0.52	−10.79 to 16.61
A febrile child should always be examined by a physician	25 (25)	14 (23)	11 (28)	0.57	−13.16 to 24.16
A febrile child should always have blood tests drawn	10 (10)	4 (7)	6 (15)	0.2	−5.39 to 23.65
A febrile child should always be treated with antibiotics	1 (1)	1 (2)	0 (0)	0.37	−7.02 to 9.48
Teething can cause fever	74 (74)	40 (67)	34 (85)	0.05	−0.83 to 34.26

* Chi-square comparison of two groups.

## DISCUSSION

Caregivers of children in this study had limited knowledge of correct fever definition, management, and complications, regardless of the type of referral that brought them to the ED. This suggests that there are still missed opportunities to teach care-givers the correct facts about fever in children and its management, both in office settings and in the ED.

Fever in children is a common problem and is alarming to parents. In humans, increased temperature is associated with decreased microbial reproduction and increased inflammatory response.^5^ It is not the fever itself but the fear of possible complications and accompanying symptoms that is important for pediatricians and parents. Parental misconceptions often lead them to unnecessarily aggressive and inappropriate management of fever in their children.^10^ The majority of the caregivers in our study were well educated, yet most were misinformed regarding many aspects of fever and its management. Despite their visit to the primary care physician’s office, the fear of brain damage still existed in similar proportions compared to those caregivers who had not seen their primary care physician prior to the ED visit.

For decades, medical professionals have tried to educate caregivers about fever, but our study, similarly to previous studies,^3^,^4^,^8^,^11^ demonstrates that a knowledge gap still exists. The basic problem of defining fever is prevalent, even in caregivers who had recently visited their primary care physician. We observed a lower rate of use of antipyretics compared to the literature (10% versus 33%–65%).^8^,^12^,^13^ Thus, it seems that caregivers’ knowledge and practices can be changed, with appropriate education.

Pediatric health care providers are uniquely situated to be able to make an impact on parental understanding of fever and its role in illness by providing clear and authoritative information.^14^ It is reasonable to expect that primary care physicians (pediatricians and family physicians) would assume the role of educating parents about basic medical realities, such as common febrile disease in children, the correct management of a febrile child, helpful remedies, and indications for seeking immediate medical advice. The fact that caregivers’ knowledge did not differ between our two groups of caregivers suggests that there is no educational benefit from the office visit to the primary care physician at the time of referral to the hospital, during acute illness. Nonetheless, it is also important to mention the possible lack of physicians’ awareness of caregivers’ misconceptions.

Our study is limited in that caregivers unable to understand the questions due to a language barrier were not approached, and caregivers with a low educational level were not well represented in our sample. Another limitation is the fact that this is a single-center study, which limits the option to generalize its conclusions.

We conclude that undue fear and overly aggressive treatment of fever are still common among caregivers of infants and young children. Overall, we observed a decrease in use of antipyretics, but we still found that caregivers’ lack of knowledge and fear of complications persist. Considerable effort will be required on the part of pediatricians to re-educate caregivers about the definition of fever and its appropriate treatment. Implementation of educational programs regarding the management of the febrile child is needed in clinics as well as in the ED.

## Abbreviations

ED: emergency department
